# Rothia similimucilaginosa sp. nov., isolated from the human nasal cavity

**DOI:** 10.1099/ijsem.0.007024

**Published:** 2026-01-16

**Authors:** Mercedes Pérez Pérez, Jacobey King, Paul A. Lawson, Reed M. Stubbendieck

**Affiliations:** 1 Department of Microbiology and Molecular Genetics, Oklahoma State University, Stillwater, OK 74078, USA; 2 School of Biological Sciences, University of Oklahoma, Norman, OK 73019, USA

**Keywords:** nasal microbiome, nasal microbiota, *Rothia*, *Rothia mucilaginosa*, *Rothia similimucilaginosa*

## Abstract

Four strains of a Gram-stain-positive, coccoid, catalase-positive, non-motile bacterium were recovered from nasal lavage samples collected from children in Wisconsin during the Spring of 2008. These strains, designated RSM42^T^, RSM292, RSM386 and RSM407, were subjected to a comprehensive biochemical and polyphasic taxonomic investigation. Despite the novel bacterium sharing 99.6% 16S rRNA gene sequence identity with *Rothia mucilaginosa* 5762/67^T^, BLAST+ average nucleotide identity, MUMmer3 average nucleotide identity and digital DNA–DNA hybridization values of 91.3%, 91.9% and 43.1%, respectively, were below the cut-off values routinely used for species demarcation. Consistent with these findings, phylogenetic and pangenomic comparisons indicated that RSM42^T^, RSM292, RSM386 and RSM407 form a separate lineage within the genus *Rothia*. Strain RSM42^T^ is further distinguished from *R. mucilaginosa* 5762/67^T^ by its unique ability among *Rothia* species to use fructose-6-phosphate as a sole carbon source. RSM42^T^ also exhibits an enzyme activity profile consistent with *R. mucilaginosa*, as it is positive for valine arylamidase and negative for C4 esterase, β-glucosidase, pyrazinamidase and trypsin, a combination not observed in other *Rothia* species. The major fatty acids were anteiso-C_15:0_ (44.2%) and iso-C_16:0_ (14.4%), and the moderate fatty acids were anteiso-C_13:0_ (2.3%), iso-C_14:0_ (6.0%), C_14:0_ (2.3%), iso-C_15:0_ (5.9%), C_15:0_ (1.9%), C_16:0_ (9.3%) and anteiso-C_17:0_ (9.5%). The major polar lipids were aminoglycolipid and diphosphatidylglycerol. Based on biochemical, phylogenetic, genotypic and chemotaxonomic criteria, these isolates represent a novel species within the genus *Rothia*, closely related to *R. mucilaginosa*, for which the name *Rothia similimucilaginosa* sp. nov. is proposed. The type strain is RSM42^T^ (=ATCC TSD-447^T^=DSM 118581^T^).

## Data Availability

GenBank accession numbers for each strain used in this study are provided in Table 1. The scripts necessary to replicate this work are available at https://github.com/reedstubbendieck/rothia_species_characterization, and all necessary tools used are provided below. The derived datasets are available at https://doi.org/10.6084/m9.figshare.28732967.

**Table 1.** Phenotypic characteristics separating strain RSM42^T^ from other *Rothia* species Strains: 1, RSM42^T^; 2, *R. mucilaginosa* 5762/67^T^; 3, *R. dentocariosa* ATCC 17931^T^; 4, *R. aeria* A1-17B^T^; 5, *Rothia amarae* J18^T^; 6, *Rothia aerolata* 140917-MRSA-09^T^; 7, *Rothia terrae* LMG 23708^T^; 8, *Rothia endophytica* YIM 67072^T^; 9, *Rothia nasimurium* CCUG3 5957^T^; 10, *Rothia halotolerans* CCTCC AB 206069^T^; 11, *Rothia kristinae* ATCC 27570^T^; 12, *Rothia uropygialis* 36^T^; 13, *Rothia uropygioeca* 257^T^; 14, *Rothia koreensis* P31^T^. All data were obtained under identical conditions, unless indicated otherwise. +, Positive, −, negative, (+), weak positive reaction; nd, no data.

Characteristic	1	2	3^*^	4^*^	5^†^	6^‡^	7^§^	8^||^
Colony morphology	Cream or white, mucoid, adherent to agar	Transparent or white, mucoid, glistening, smooth, convex, adherent to agar	Mature colonies may be heaped and rough or smooth	Cream or white, young colonies are smooth, old colonies are rough, adherent to agar	Cream, mucoid, smooth, rough when older	Cream or white, non-translucent	Cream or white, circular, convex, entire edges	Cream or yellow, smooth, rough when older
Cell morphology	Gram-stain-positive, coccoid, occur in pairs or clusters, diameter: 1.0–1.8 µm	Gram-stain-positive, pairs or clusters, diameter: 0.9–1.6 µm	Gram-stain-positive, coccoid, diphtheroid, filamentous or mycelial	Gram-stain-positive, coccoid, cocco-bacillary or filamentous	Gram-stain-positive, ovoid, pairs, tetrads or clusters, diameter: 0.6–0.9 µm	Gram-stain-positive, coccoid	Gram-stain-positive, ovoid to spherical, occur singly or in pairs or tetrads, diameter 1.0–2.0 µm	Gram-stain-positive, ovoid to spherical, occur singly or in pairs or tetrads, diameter 1.0–2.0 µm
Growth temperature range	30–37 °C (weak at 25, 40 or 45 °C)	30–37 °C (weak at 25 or 40 °C)	nd	nd	30–37 °C	16–36 °C (weak at 42 °C)	15–40 °C	4–45 °C
Growth pH range	6–8 (weak at 9–10)	6–8	nd	nd	nd	5–10	5–10	5–10
NaCl tolerance (range, %)	0.5–4.5% (weak at 5.5%)	0.5–4%	nd	nd	nd	0–7%	0–7%	0–7%
DNA G+C content (mol%)	58.4	59.5	53.7	56.8	52.2	57.9	53.4	56.3
Enzyme activities:								
C4 esterase	−	−	nd	nd	nd	−	+	−
β-Glucosidase	−	−	+	+	nd	−	+	+
Pyrazinamidase	−	−	nd	nd	nd	+	+	+
Trypsin	−	−	−	nd	−	−	−	+
Valine arylamidase	+	+	−	nd	(+)	−	−	+
Reactions:								
Gelatin hydrolysis	+	+	nd	nd	+	+	+	+
Nitrate reduction	+	+	nd	+	+	+	+	+
Accession	GCA_029850985.1	6d106fed56314878¶	GCA_000164695.2	GCA_002355935.1	GCA_039525765.1	GCA_014635585.1	GCA_012396615.1	GCA_039543885.1

**Characteristic**	**9** ^**#**^	**10** ^**^	**11††**	**12‡‡**	**13‡‡**	**14§§**
Colony morphology	Non-pigmented, α-haemolytic	Pale yellow, circular, opaque, slightly convex, smooth	Pale cream to pale orange, small, highly convex when young	Pale orange, convex, circular, smooth, shiny, γ-haemolytic	Pale orange, convex, circular, smooth, shiny, γ-haemolytic	Pale cream to pale orange, circular, smooth, opaque
Cell morphology	Gram-stain-positive, cocci	Gram-stain-positive, coccoid, diameter: 0.6–1 µm	Gram-stain-positive, spheres, tetrads and clusters, diameter: 0.7–1.1 µm	Gram-stain-positive cocci	Gram-stain-positive cocci	Gram-stain-positive, coccoid, diameter: 1.0–1.5 µm
Growth temperature	nd	10–37 °C	nd	25–40 °C	25–40 °C	15–37 °C
Growth pH	nd	6–9	nd	4–9	4–9	5–10
NaCl tolerance (range, %)	nd	0–10%	10%	0–15%	0–15%	0–15%
DNA G+C content (mol%)	59.9	71.5	71.9	61.1	60.8	64.5
Enzyme activities:						
C4 esterase	−	−	nd	+	+	+
β-Glucosidase	−	+	nd	−	+	+
Pyrazinamidase	+	+	nd	nd	nd	nd
Trypsin	+	+	nd	−	−	−
Valine arylamidase	+	+	nd	−	−	−
Reactions:						
Gelatin hydrolysis	nd	−	nd	+	+	−
Nitrate reduction	+	+	−	−	−	−
Accession	GCA_042660305.1	GCA_004136635.1	GCA_004136565.1	GCA_004137765.1	GCA_004136585.1	GCA_004136575.1

*Data from [7], cultivated on BHI.

†Data from [10], cultivated on blood agar.

‡Data from [54].

§Data from [8].

||Data from [14].

¶Available from the ATCC Website: https://genomes.atcc.org/genomes/6d106fed56314878.

#Data from [11], cultivated on blood agar.

**Data from [9], cultivated on glycerol asparagine agar with 5% NaCl.

††Data from [59].

‡‡Data from [60], cultivated on Columbia agar supplemented with 5% sheep blood and Müller–Hinton agar.

§§Data from [61], cultivated on Marine agar.

## Introduction

Initially, the genus *Rothia* was classified as *Nocardia* in the family *Actinomycetaceae* based on the morphological characteristics of its members [1]. These bacteria were later renamed *Rothia* by Georg and Brown [2] and then transferred to the family *Micrococcaceae* based on phylogenetic inferences by Stackebrandt *et al*. [3]. Presently, there are 20 *Rothia* species listed in the List of Prokaryotic Names with Standing in Nomenclature [4]. However, five of these species are not validly published, and two have been identified through metagenomic sequencing of the chicken gut, but have not been cultured [5]. *Rothia* normally presents as Gram-stain-positive, coccoid cells. Colonies are often adherent, beige or cream in colour, and their morphology varies. The colonies of some species are described as raised and smooth, while others are crumbly in appearance [6]. The natural habitats of *Rothia* are broad and include environmental sources (e.g. air [7], soil [8,9] and waste material [10]), as well as host-associated sources (e.g. the oral and respiratory tracts of humans and other animals [2,11,13] and plants [14]). In adults sampled as part of the Human Microbiome Project [15], *Rothia* were found at relative abundances of 1.1–12.6% in the oral tract, but are typically found at ≤0.50% in the anterior nares and nasal cavity [16,19]. Within hosts, *Rothia* are frequently involved in microbe–microbe and host–microbe interactions [20]. Though prior research has focused largely on the potential of *Rothia* spp. as opportunistic pathogens [21], these bacteria can also provide benefits to human hosts. For instance, *Rothia mucilaginosa* inhibits lipopolysaccharide-induced inflammation in an alveolar epithelial cell model and in murine lungs infected with *Pseudomonas aeruginosa*. This microbe is also inversely associated with pro-inflammatory markers *in vivo* [22]. Further, *Rothia* spp. are more abundant in the nasal cavities of healthy children and can secrete a peptidoglycan endopeptidase called secreted antigen A (SagA), which inhibits the growth of the nasal pathobiont *Moraxella catarrhalis* [23]. *Rothia* encode biosynthetic gene clusters (BGCs) for the production of antibiotics and other secondary metabolites [13,19, 24, 25], but, with the exception of enterobactin from *R. mucilaginosa* [26], no bioactive compounds from these organisms have been isolated and characterized. Together, these examples highlight that *Rothia* are underexplored members of the human microbiota, which may act as mutualists, and possess potential for the development of novel therapeutics.

In the present study, we describe the characterization of four strains that represent a novel species: RSM42^T^, RSM292, RSM386 and RSM407, which were isolated from the nasal specimens of four different children in Wisconsin during the Spring of 2008.

## Isolation and ecology

During a previous investigation [23], organisms from nasal lavage specimens donated by children were cultured on brain heart infusion [BHI; 0.6% (wt/vol) brain heart infusion, 0.6% (wt/vol) peptic digest of animal tissue, 0.5% sodium chloride (NaCl), 0.3% (wt/vol) dextrose, 1.45% (wt/vol) pancreatic digest of gelatin, 0.25% (wt/vol) disodium phosphate; Dot Scientific] agar [BHI with 1.5% (wt/vol) agar] plates aerobically for 7 days at 37 °C. Morphologically distinct colonies were selected and subcultured to purity, and isolates were identified via colony PCR and Sanger sequencing of the 16S rRNA gene using the universal 27F and 1492R primers [27]. In total, 14 *Rothia* isolates were cultured (six *Rothia aeria*, four *Rothia dentocariosa* and four of a novel *Rothia* species) [23].

## Phylogeny and genomic features

Genomic DNA from isolates of interest was isolated using either the DNA Miniprep kit (ZymoBIOMICS) with 2×5 min bead-beating steps or the MasterPure Yeast DNA Purification Kit (Lucigen) with the addition of 1 µl of Ready-Lyse Lysozyme Solution (Lucigen) and 1 µl of 5 mg ml^−1^ RNase A (Lucigen). DNA libraries were prepared with the Illumina Nextera Kit and sequenced using the 2×150 bp paired-end Illumina NextSeq 2000 platform at SeqCenter. Raw reads were processed using fastp V0.2.0 [28] with default parameters, which discard bases with Phred quality scores <15, discard reads shorter than 15 bp and remove reads with >40% low-quality bases or >5 N bases. Draft genomes were assembled using SPAdes V3.11.1 [29] with default parameters. To generate a complete genome sequence for strain RSM42^T^, genomic DNA was extracted as described above, and transposome-complex, amplification-free long-read sequencing libraries were prepared and sequenced using Oxford Nanopore Technology at Plasmidsaurus. The draft and complete genome sequences were previously deposited under BioProject accession number PRJNA867425. The genome assembly statistics and accessions for all strains used in this study are provided in Table S1 (available in the online Supplementary Material).

**Fig. 1.**
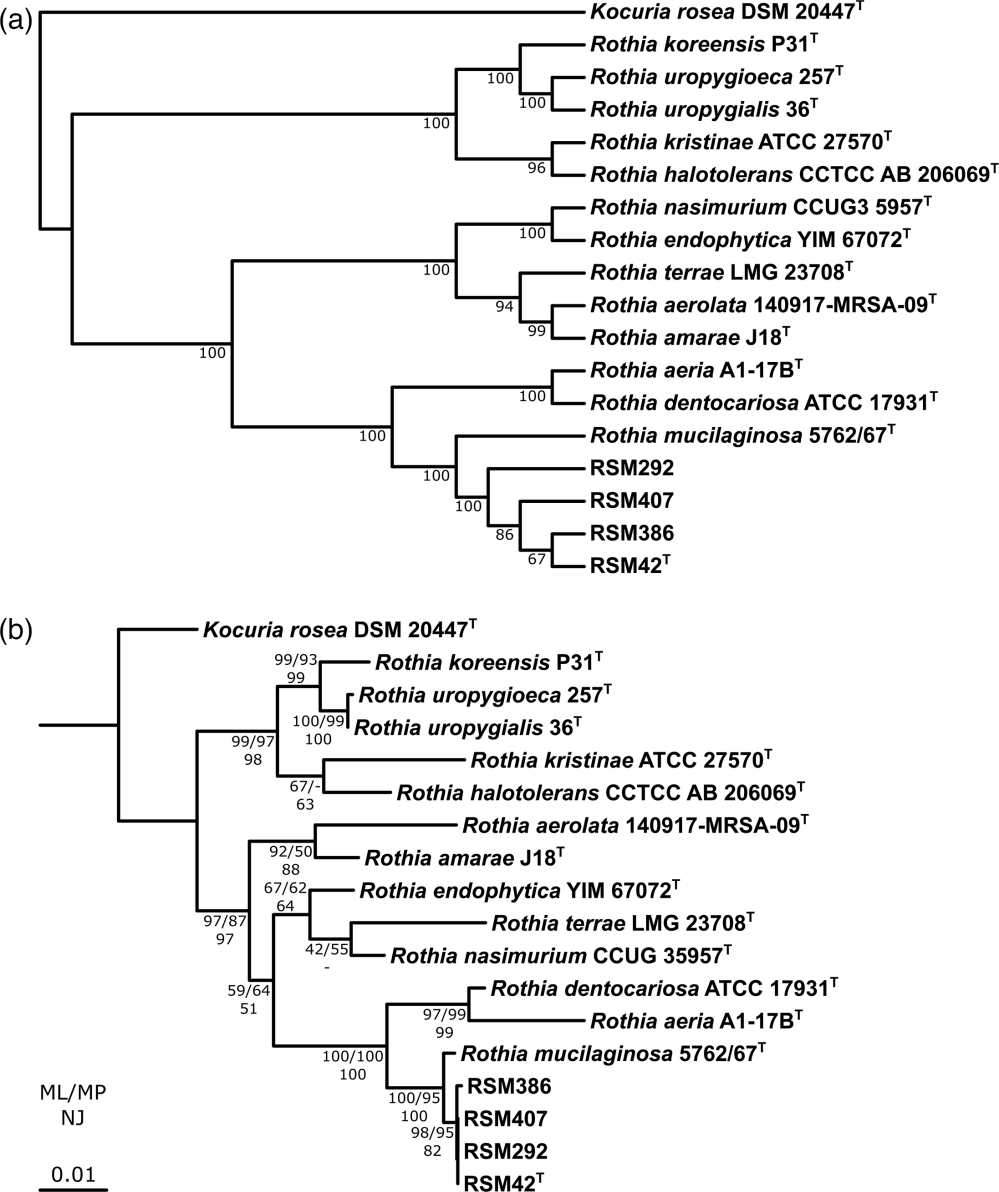
Phylogeny of *Rothia*. (**a**) Core genome phylogeny showing the relationships between RSM42^T^, RSM292, RSM386, RSM407 and closely related strains of *Rothia*. The phylogenetic tree was constructed using 93 conserved single-copy bacterial genes (GenProp0799). The phylogeny is rooted on *Kocuria rosea* DSM 20477^T^, and branch lengths were transformed to be proportional to the root. Bootstrap support (100 replicates) is indicated at the nodes. (**b**) ML16S rRNA gene phylogeny showing the relationships between RSM42^T^, RSM292, RSM386, RSM407 and closely related strains of *Rothia*. The phylogeny is rooted on *K. rosea* DSM 20477^T^. Branch lengths indicate the number of substitutions per site, as indicated by the scale bar. Bootstrap support (1,000 replicates) at each node is indicated with the corresponding percentage for ML, MP and NJ trees.

The core genome phylogeny of these four *Rothia* strains and all other validly published *Rothia* species was constructed using core_species_tree.pl (https://github.com/chevrm/core_species_tree) [30]. This approach uses Prodigal V2.6.3 [31] for gene calling, identifies 93 conserved single-copy bacterial genes (GenProp0799) from each genome using hmmscan from HMMER V3.3.2, performs amino acid alignments of these genes and then converts those alignments into codon alignments. Subsequently, gene trees are generated for each alignment using RaxML V8.2.12 with the following parameters: -m GTRGAMMA -f a -x 897543 -n 100 -p 345232. After this initial run, RaxML is used to place bootstrap values on the best-scoring maximum-likelihood (ML) tree with the following parameters: -f b -m GTRGAMMA. The individual gene trees and bootstrap values are then combined into a species tree using a coalescent-based approach with ASTRAL V5.7.8 [32] with the following parameter: -r 100. In this phylogeny, all four strains formed a monophyletic clade, which was distinct, but closely related to *R. mucilaginosa* (Fig. 1a). Next, the 16S rRNA gene sequences were extracted from these *Rothia* genomes using the ContEst16S webserver (https://www.ezbiocloud.net/tools/contest16s) [33], and mega11 [34] was used to perform alignments using the MUSCLE algorithm with neighbour-joining (NJ) and a gap-opening penalty of −400. mega11 was then used to construct ML (general time-reversible [GTR] model, six gamma categories, invariant sites), maximum parsimony (MP) and NJ (maximum composite likelihood, uniform rates) trees, all with complete gap deletion and 1,000 bootstrap replicates for support. A similar tree topology was obtained using the 16S rRNA gene sequence alone, when compared with the core genome phylogeny, though bootstrap support values for the former were lower, particularly for the shallower branches (Fig. 1b).

Using pyani V0.2.12 [35], the average nucleotide identity (ANI) for all possible pairwise *Rothia* combinations was calculated using BLAST+ ANI (ANIb) and MUMmer3 ANI (ANIm), both with default parameters. Strains RSM42^T^, RSM292, RSM386 and RSM407 did not reach the 95% ANI threshold commonly used to delineate species [36] with *R. mucilaginosa* 5762/67^T^ (ANIb: 91.3–91.4%, coverage: 87.1–90.0%; ANIm: 91.9–92%, coverage: 90.6–92.6%) (Tables S2 and S3) [11]. Further, the digital DNA–DNA hybridization (dDDH) values determined using the Genome-to-Genome Distance Calculator 3.0 webserver (https://ggdc.dsmz.de/ggdc.php) [37] demonstrated that none of these four strains reached the 70% dDDH threshold commonly used to delineate species [36] with *R. mucilaginosa* 5762/67^T^ (dDDH: 43.1–43.5%) (Table S4). Therefore, both ANI and dDDH reinforced the separated species status between the novel organism and *R. mucilaginosa*.

Bakta V1.8.1 [38] was used to predict open-reading frames in the genomes using default parameters, and then the AAI-profiler webserver (http://ekhidna2.biocenter.helsinki.fi/AAI/) [39] was used to determine the average amino acid identity (AAI) of the corresponding protein sequences between these strains and other *Rothia*. Strains RSM42^T^, RSM292, RSM386 and RSM407 exhibit strong AAI with *R. mucilaginosa* (AAI: 95.7–95.8%, matched fraction: 93.0–94.4%). These findings are consistent with previous phylogenetic and pangenomic analyses, indicating a close relationship between these strains and *R. mucilaginosa* [23].

**Fig. 2.**
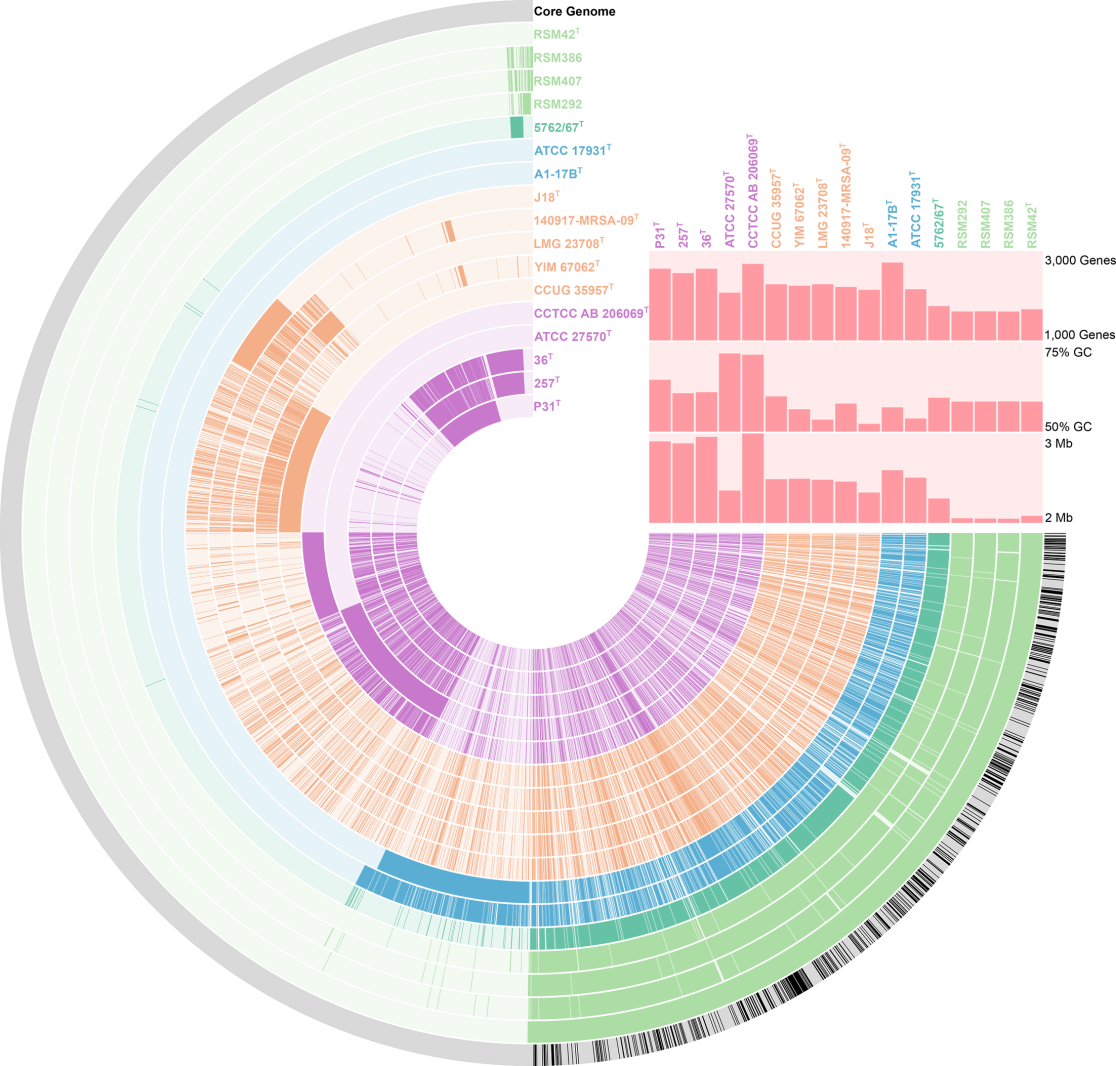
Pangenome of *Rothia*. This pangenome was generated from the genome sequences of the 13 type species of *Rothia* and four draft genome sequences of the strains used in this study, using the anvi’o 8 pangenome pipeline. Each arc represents the genetic content of a single genome ordered based on relative order in RSM42^T^. The position of each arc is based on the phylogenetic position in Fig. 1a. In total, this pangenome contains 30,521 genes within 4,288 gene clusters. The presence or absence of a gene cluster within a given genome is indicated by opaque and transparent colours, respectively. The black arc indicates the set of single-copy core genes. The top right indicates total predicted genes (top), G+C content (middle) and genome size (bottom).

The pangenome of RSM42^T^, RSM292, RSM386 and RSM407, along with all other validly published *Rothia* species, was generated using the anvi’o 8 pangenomics pipeline [40] with the following parameters: --minbit 0.5 --mcl-inflation 10 --min-occurrence 2. Significant differences in the genomes of the different *Rothia* species were observed, which were consistent with the clades present within the core genome phylogeny (Fig. 2). This pangenome demonstrates that RSM42^T^, RSM292, RSM386 and RSM407 have similar gene counts, overall core and accessory gene content, and genomic G+C content when compared with *R. mucilaginosa* 5762/67^T^, though the latter possesses a genome that is ~200 kbp larger than the others. The anvi-run-kegg-kofams programme was used to perform an hmmscan against the KofamKOALA [41] to annotate proteins with Kyoto Encyclopaedia of Gene and Genomes (KEGG) database terms [42]. Subsequently, the anvi-estimate-metabolism programme was used to reconstruct metabolism for modules in the KEGG MODULE database [43] and estimates their completeness using a pathwise approach. Hierarchical clustering of the presence–absence of these KEGG modules largely mirrors the phylogenetic relationships among *Rothia* species (Fig. S1). Compared with other *Rothia*, the human-associated species (i.e., *R. aeria*, *R. dentocariosa*, *R. mucilaginosa* and the novel species) lack complete modules for central carbon metabolism (e.g., Krebs cycle), amino acid biosynthesis (e.g., arginine, histidine, proline), sulphur assimilation, sugar interconversion, vitamin salvage and purine degradation pathways. Further, RSM42^T^, RSM292, RSM386 and RSM407 each possess the ‘Methionine biosynthesis’ module (M00017), which was lacking in *R. mucilaginosa* 5762/67^T^. Otherwise, there were no differences in their KEGG module content.

Using multismash [44], each *Rothia* genome was examined for secondary metabolite BGCs using antiSMASH 7 [45], and BiG-SCAPE v1.15 [46] to cluster these BGCs into gene cluster families, both with default parameters. Consistent with previous results, strains RSM42^T^, RSM292, RSM386, RSM407 and *R. mucilaginosa* 5762/67^T^ each encode a single BGC for the siderophore enterobactin [23,26] (Fig. S2a). Analysis of these BGCs with clinker V0.0.28 [47] showed that the enterobactin BGC was highly conserved across these strains (Fig. S2b). However, under laboratory conditions, in our hands, siderophore production by RSM42^T^ was previously not observed using a chrome azurol S assay [23]. No other *Rothia* species encoded this BGC.

Together, these data suggest that these four strains are closely related to, albeit distinct from, *R. mucilaginosa*.

## Physiology and chemotaxonomy

Phenotype investigations of strains RSM42^T^, RSM292, RSM386, RSM407 and *R. mucilaginosa* 5762/67^T^ were performed. All strains were cultured on tryptone soy broth [TSB; 1.7% (wt/vol) pancreatic digest of casein, 0.3% (wt/vol) papaic digest of soybean, 0.25% (wt/vol) dextrose, 0.5% (wt/vol) NaCl, 0.25% (wt/vol) dipotassium phosphate; BD] or tryptone soya agar [TSA; TSB with 1.5% (wt/vol) agar] plates at 37 °C for 2–3 days. A Nikon Eclipse Ni-E epifluorescent microscope equipped with a 100×/1.45 NA objective and Zyla 4.2 Plus camera was used for all microscopic observations. Standard methods for Gram-staining and the hanging-drop method were used to assess cell morphology and motility [48]. Each of the four strains had a coccoid morphology, with cell sizes between 1.0 and 1.8 µm, was Gram-stain-positive and was non-motile (Table 1).

**Fig. 3.**
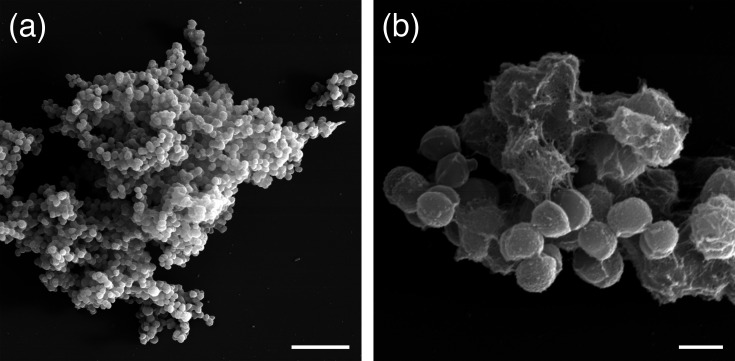
Scanning electron micrographs of clusters of RSM42^T^. (**a**) Scale bar is 10 µm. (**b**) Scale bar is 1 µm.

Scanning electron microscopy was used to examine the cell surface structure of strain RSM42^T^ at high detail. The strain was cultured aerobically in 3 ml TSB for 24 h. Following incubation, 100 µl of culture was centrifuged at 21,100***g*** for 5 min at ambient temperature and washed with PBS (Gibco). The cells were then fixed in 2.0% (wt/vol) glutaraldehyde in 0.1 M sodium cacodylate buffer for 2 h at ambient temperature. After fixation, samples were washed three times with 0.1 M sodium cacodylate containing 0.35 M sucrose. The sample was adhered to a coverslip coated with 1 µg ml^−1^ high molecular weight poly-l-lysine and post-fixed with 1% (wt/vol) osmium tetroxide for 1 h at ambient temperature. The sample was again washed three times, dehydrated through a graded ethanol series and subjected to two washes in hexamethyldisilazane. Finally, the sample was sputter-coated with a thin layer of gold–palladium (Au–Pd) alloy to enhance surface conductivity and examined using a field-emission scanning electron microscope (Scios 2 LoVac SEM; Thermo Fisher) at an acceleration voltage of 20 kV at the Oklahoma State University Microscopy Facility. Strain RSM42^T^ was confirmed to possess coccoid morphology and occur in clusters containing tens to thousands of cells (Fig. 3a). Thin filamentous strands were observed on the surface and between cells, suggesting that these bacteria may adhere together through production of extracellular polymeric substances. However, the composition of these strands is currently unknown (Fig. 3b).

Growth of RSM42^T^, RSM292, RSM386, RSM407 and *R. mucilaginosa* 5762/67^T^ was assessed visually for colony formation at 4, 10, 15, 20, 25, 30, 35, 37, 40 and 45 °C on TSA after 2 days. The strains grew well at temperatures between 30 and 37 °C (Table 1). To assess growth under anaerobic conditions, the strains were cultured on TSA, incubated at 37 °C in a GasPak Jar (BD) and observed as above. A minimal number of small (<0.5 mm) colonies were observed for each strain under anaerobic conditions. In all cases, dry anaerobic strips (BD) confirmed that the environment inside the GasPak jar was anaerobic. Tolerance to NaCl [0.5–10.5% (wt/vol), in 1% increments) and the ability to grow at various pH levels (pH 4–10, in 1-unit increments) was evaluated using the buffer system described by Xu *et al*. [49]. Note that the basal TSB medium contains 0.5% (wt/vol) NaCl. For these tests, a single colony was inoculated into 3 ml TSB with the specified NaCl concentration or buffered to the given pH, and growth was assessed visually after incubating the tubes for 2 days while shaking at 37 °C. The strains grew well in a pH range of 6–8 and weakly with scant, flocculent growth at pH 9 and 10 (Table 1). In addition, the strains grew at NaCl concentrations of 0.5–4.5% (wt/vol), and weakly with scant, flocculent growth at 5.5% (wt/vol), but no growth was observed at 6.5% (wt/vol) and above (Table 1).

Strains RSM42^T^, RSM292, RSM386, RSM407 and *R. mucilaginosa* 5762/67^T^ were then examined for a range of biochemical activities using API Coryne (bioMérieux) and API ZYM (bioMérieux) kits and for sole carbon source utilization using GP2 microplates (Biolog), following the manufacturer’s instructions. In addition, using standard methods with 3% (wt/vol) hydrogen peroxide, catalase production was confirmed for RSM42^T^, RSM292, RSM386 and RSM407, consistent with all other *Rothia* species. The distinguishing traits for RSM42^T^ are provided in Tables1  2. The results for RSM292, RSM386 and RSM407 are provided in Tables S5 and S6. RSM42^T^ is positive for valine arylamidase and negative for C4 esterase, β-glucosidase, pyrazinamidase and trypsin, which distinguishes it and *R. mucilaginosa* from other *Rothia* species. RSM42^T^ also showed gelatin hydrolysis and nitrate reduction, consistent with the most closely related species. Utilization of d-fructose-6-phosphate as a sole carbon source further differentiated RSM42^T^, RSM292, RSM386 and RSM407 from all other *Rothia*. In contrast to *R. mucilaginosa* 5762/67^T^, RSM42^T^ did not utilize d-salicin or β-methyl-d-glucoside as sole carbon sources. However, RSM292, RSM386 and RSM407 were able to utilize these carbon sources (Table S6).

**Table 2.** Carbon source utilization by RSM42^T^ and other *Rothia* species Strains: 1, RSM42^T^; 2, *R. mucilaginosa* 5762/67^T^; 3, *R. dentocariosa* ATCC 17931^T^; 4, *R. aeria* A1-17B^T^; 5, *Rothia amarae* J18^T^; 6, *Rothia aerolata* 140917-MRSA-09^T^; 7, *Rothia terrae* LMG 23708^T^; 8, *Rothia endophytica* YIM 67072^T^; 9, *Rothia nasimurium* CCUG3 5957^T^*;* 10, *Rothia halotolerans* CCTCC AB 206069^T^; 11, *Rothia kristinae* ATCC 27570^T^; 12, *Rothia uropygialis* 36^T^; 13, *Rothia uropygioeca* 257^T^; 14, *Rothia koreensis* P31^T^. All data were obtained under identical conditions, unless indicated otherwise. +, Positive; −, negative; (+), weak positive reaction. nd, no data.

Carbon source	1	2	3^*^	4^*^	5^†^	6^‡^	7^§^	8^||^	9^¶^	10 #	11 **	12^††^	13^††^	14 ‡‡
Dextrin	+	+	−	+	nd	nd	+	−	−	nd	nd	nd	nd	−
d-Maltose	+	+	+	+	+	+	+	+	+	+	+	+	+	−
d-Trehalose	+	+	+	+	+	+	+	−	+	−	nd	nd	nd	nd
d-Turanose	+	+	+	+	nd	nd	+	−	−	+	nd	nd	nd	−
β-Methyl-d-glucoside	−	+	−	+	nd	−	−	−	+	−	nd	nd	nd	nd
d-Salicin	−	+	−	+	+	−	−	−	−	+	nd	nd	nd	nd
α-d-Glucose	+	+	+	+	+	+	+	−	+	+	+	−	−	+
d-Mannose	+	+	+	+	+	+	+	−	nd	nd	+	+	+	+
d-Fructose	+	+	+	+	nd	+	+	−	nd	+	+	nd	nd	+
d-Galactose	−	(+)	−	−	nd	+	−	−	−	+	(+)	nd	nd	nd
3-Methyl glucose	−	−	−	+	nd	nd	+	−	+	nd	nd	nd	nd	−
d-Serine	−	−	−	−	nd	+	+	−	nd	nd	nd	nd	nd	nd
d-Fructose-6-phosphate	+	−	−	−	nd	nd	−	−	−	−	nd	nd	nd	nd
Gelatin	−	−	nd	nd	+	−	+	+	−	−	nd	+	+	−
Glycyl-l-proline	+	+	nd	nd	nd	nd	−	−	nd	nd	nd	nd	nd	nd
l-Serine	+	−	−	−	nd	+	+	−	nd	nd	nd	nd	nd	nd
Pectin	+	+	nd	nd	nd	nd	−	−	−	−	nd	nd	nd	nd
d-Galacturonic acid	+	+	−	−	nd	nd	−	−	+	−	nd	nd	nd	−
d-Glucuronic acid	−	−	nd	nd	nd	nd	−	−	−	−	nd	nd	nd	−
d-Lactic acid methyl ester	+	+	−	−	nd	nd	+	−	nd	−	nd	nd	nd	nd
l-Lactic acid	+	+	+	+	nd	+	+	−	−	−	nd	nd	nd	+
α-Keto-butyric acid	+	+	+	+	nd	+	−	−	−	−	nd	nd	nd	nd
Acetoacetic acid	+	−	nd	nd	nd	nd	−	−	−	−	nd	nd	nd	nd
Acetic acid	−	−	−	−	nd	−	−	−	−	−	nd	nd	nd	+

*Data from [7].

†Data from [10], cultivated on blood agar.

‡Data from [54].

§Data from [8].

||Data from [14].

¶Data from [11], cultivated on blood agar.

#Data from [9], cultivated on glycerol asparagine agar with 5% NaCl.

**Data from [59].

††Data from [60].

‡‡Data from [61].

The Comprehensive Antibiotic Resistance Database Resistance Gene Identifier (CARD rgi; https://card.mcmaster.ca/analyze/rgi) [50] detected multiple loose hits to putative antibiotic resistance genes in RSM42^T^, RSM292, RSM386 and RSM407, including a single nucleotide polymorphism in a homologue of *gidB* that results in a V124G substitution, which results in streptomycin resistance in *Mycobacterium tuberculosis* [51], and several hits to putative ATP-binding cassette and resistance–nodulation–cell division efflux pumps. In addition, CARD rgi detected a single strict hit to the *vanY* gene for glycopeptide resistance in RSM42^T^ (Table S7).

Antimicrobial susceptibility testing was performed for strains RSM42^T^ and *R. mucilaginosa* 5762/67^T^ following the broth microdilution protocol and standards from the Clinical and Laboratory Standards Institute (CLSI) [52]. The following antibiotics were tested: ampicillin (OmniPur), erythromycin (Thermo Fisher Scientific), gentamicin (Acros Organics), kanamycin (Thermo Fisher Scientific), rifampicin (Thermo Fisher Scientific), spectinomycin (MP Biomedicals), streptomycin (Acros Organics) and vancomycin (Thermo Fisher Scientific). For quality control, strains *Staphylococcus aureus* subsp. *aureus* strain Wichita and *Escherichia coli* FDA strain Seattle 1946 were included as controls, which behaved as expected. Based on the CLSI MIC breakpoints for *R. mucilaginosa*, strain RSM24^T^ and *R. mucilaginosa* 5762/67^T^ are both sensitive to erythromycin (MIC [µg ml^−1^] ≤0.5) and vancomycin (MIC [µg ml^−1^] ≤2) [53]. The latter result is inconsistent with the results from the CARD rgi, which detected a *vanY* homologue encoded by RSM42^T^ (Tables 3 and S7). Specific breakpoints are not reported for the other antibiotics tested. Strain RSM42^T^ was more resistant to gentamicin, kanamycin, spectinomycin and streptomycin than *R. mucilaginosa* 5762/67^T^ (Table 3).

**Table 3.** Antimicrobial susceptibility test for RSM42^T^ and *R. mucilaginosa* 5762/67^T^ Strains: 1, RSM42^T^; 2, *R. mucilaginosa* 5762/67^T^. All data were obtained under identical conditions. The MIC values (µg ml^−1^) are reported as the lowest concentration that completely inhibits growth.

Antibiotic	1	2
Ampicillin	<0.125	<0.125
Erythromycin	<0.06	<0.06
Gentamicin	8	2
Kanamycin	32	8
Rifampicin	<0.125	<0.125
Spectinomycin	4	2
Streptomycin	2	1
Vancomycin	0.5	0.5

Analysis of the fatty acid profile for strains RSM42^T^, RSM292, RSM386, RSM407 and *R. mucilaginosa* 5762/67^T^, cultured on TSA at 30 °C for 3 days, was carried out by DSMZ Services, Leibniz-Institut DSMZ – Deutsche Sammlung von Mikroorganismen und Zellkulturen GmbH, Braunschweig, Germany. The fatty acid profiles of the four strains were composed primarily of iso- and anteiso-branched fatty acids (e.g. anteiso-C_13:0_, iso-C_14:0_, anteiso-C_15:0_, iso-C_16:0_, anteiso-C_17:0_, iso-C_15:0_ and iso-C_14:0_) and the straight-chain fatty acids C_14:0_ and C_16:0_ (Table 3). The overall fatty acid composition of these strains was similar to *R. mucilaginosa* 5762/67^T^ and consistent with other *Rothia* (Table 4). The results for RSM292, RSM386 and RSM407 are provided in Table S8.

**Table 4.** Cellular fatty acid profiles of strain RSM42^T^ and other *Rothia* species Fatty acids are listed in order of increasing retention time. Strains: 1, RSM42^T^; 2, *R. mucilaginosa* 5762/67^T^; 3, *R. dentocariosa* ATCC 17931^T^; 4, *R. aeria* A1-17B^T^; 5, *Rothia amarae* J18^T^; 6, *Rothia aerolata* 140917-MRSA-09^T^; 7, *Rothia terrae* LMG 23708^T^; 8, *Rothia endophytica* YIM 67072^T^; 9, *Rothia nasimurium* CCUG3 5957^T^; 10, *Rothia halotolerans* CCTCC AB 206069^T^; 11, *Rothia kristinae* ATCC 27570^T^; 12, *Rothia uropygialis* 36^T^; 13, *Rothia uropygioeca* 257^T^; 14, *Rothia koreensis* P31^T^. All data were obtained under identical conditions, unless indicated otherwise. −, Fatty acids with <0.5% relative abundance. nd, no data. Fatty acids with ≥10% relative abundance are shown in bold.

Fatty acid	1	2	3^*^	4^†^	5^‡^	6^§^	7^||^	8^||^	9^||^	10^¶^	11	12^#^	13^#^	14^**^
Anteiso-C_13:0_	2.3	1.3	nd	nd	−	0.5	−	2.9	2.5	nd	nd	nd	nd	nd
Iso-C_14:0_	6.0	7.6	4.0	2.7	1.5	0.6	−	2.5	5.5	1.4	nd	1.3	1.3	−
C_14:0_	2.3	1.6	nd	nd	−	0.6	1.1	1.9	0.6	1.3	nd	0.7	−	−
Iso-C_15:0_	5.9	4.7	3.3	4.5	5.5	1.6	1.2	6.2	1.7	1.1	nd	1.0	0.8	1.6
Anteiso-C_15:0_	**44.2**	**37.5**	**53.3**	**52.9**	**72.3**	**51.2**	**71.0**	**66.1**	**68.5**	**47.7**	nd	**64.8**	**56.5**	**42.9**
C_15:0_	1.9	−	nd	nd	2.5	nd	nd	nd	nd	nd	nd	nd	nd	−
Iso-C_16:0_	**14.4**	**21.2**	**14.6**	**20.3**	5.4	**10.8**	2.7	5.4	**12.2**	**15.7**	nd	**14.0**	**18.6**	**14.9**
C_16 : 0_	9.3	**10.2**	6.0	1.9	2.9	6.5	2.6	−	1.2	6.2	nd	1.7	1.4	2.8
Iso-C_17 : 0_	0.9	1.1	nd	nd	nd	0.7	0.5	−	−	nd	nd	nd	nd	−
Anteiso-C_17 : 0_	9.5	**12.5**	**16.9**	**16.0**	**10.0**	**26.8**	**14.0**	5.6	3.0	**22.1**	nd	**16.6**	**19.2**	**35.9**

*Data from [11].

†Data from [7].

‡Data from [10], cultivated on blood agar.

§Data from [54].

||Data from [14].

¶Data from [9].

#Data from [60].

**Data from [61], cultivated on marine agar.

An *in silico* analysis using KEGG was performed to investigate glycerophospholipid metabolism via the KEGG Automatic Annotation Server (https://www.genome.jp/tools/kaas/). These results indicate that RSM42^T^ encodes the enzyme phosphatidylglycerophosphate synthase (E.C. 2.7.8.5), required to synthesize phosphatidylglycerol (PG) from the essential building block cytidine diphosphate diacylglycerol. In addition, RSM42^T^ encodes the enzyme cardiolipin synthase (E.C.2.7.8.41), required to produce diphosphatidylglycerol (DPG; cardiolipin) (Fig. S3). Therefore, the major polar lipids predicted to be produced by RSM42^T^ are PG and DPG; identical results were obtained with *R. mucilaginosa* and *R. dentocariosa* (data not shown). These results were consistent with those of other close relatives of the genus *Rothia* [7,10, 54], but specifically with *R. mucilaginosa* and *R. dentocariosa*, the closest relatives of RSM42^T^ [55].

**Fig. 4.**
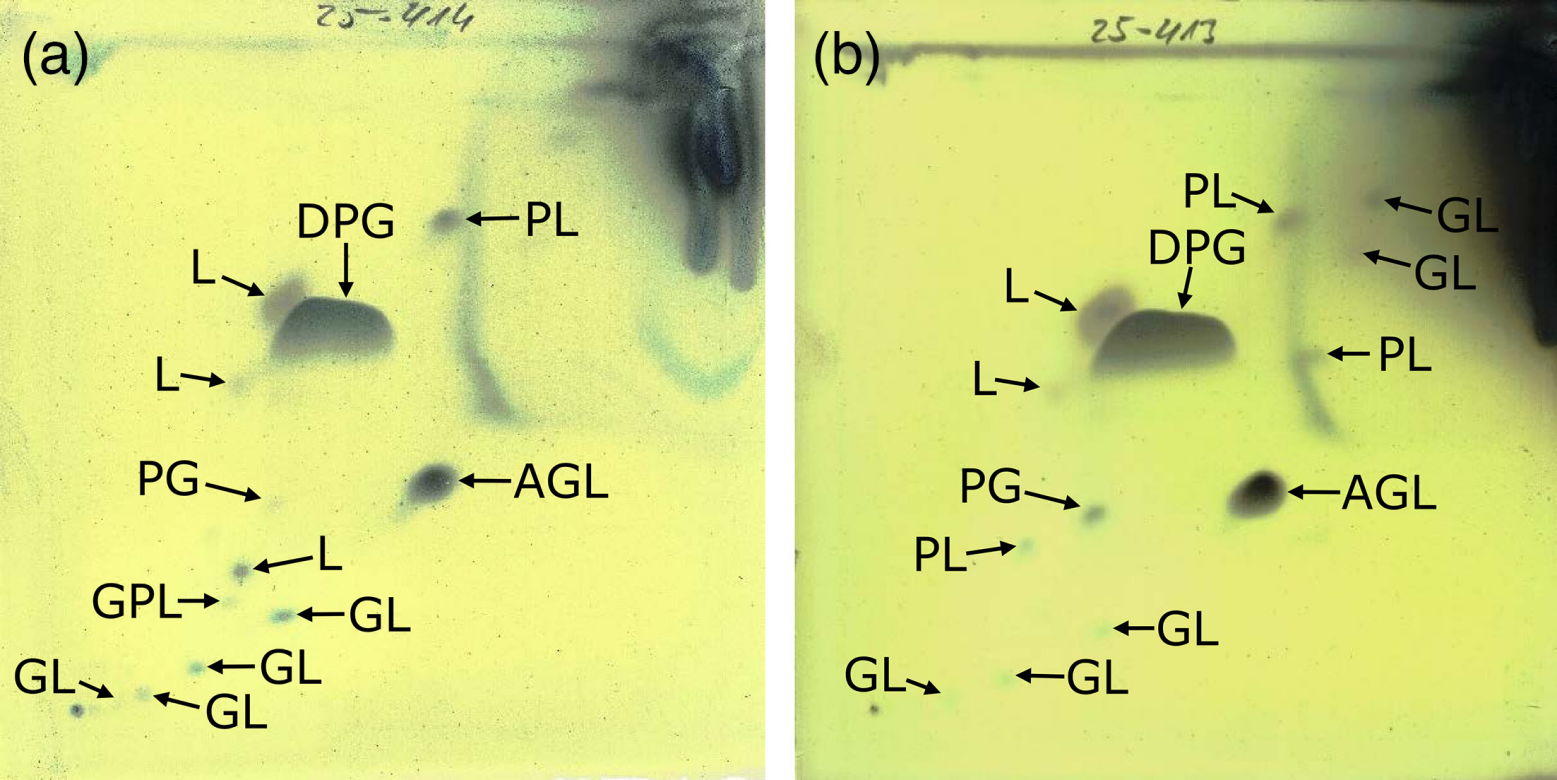
Polar lipid profile of (**a**) strain RSM42^T^ and (**b**) *R. mucilaginosa* 5762/67^T^ after two-dimensional silica gel TLC with chloroform:methanol:water in the first direction and chloroform:methanol:acetic acid:water in the second direction, with detection using molybdatophosphoric acid. Specific functional groups were detected using spray reagents and are labelled. GL, glycolipid; GPL, glycophospholipid; L, unidentified lipid; PL, unidentified phospholipid.

These *in silico* polar lipid predictions were then validated experimentally. Strain RSM42^T^ and *R. mucilaginosa* 5762/67^T^ were cultured in 25 ml TSB at 37 °C for 2 days. Cells were collected by centrifugation at 4,000***g*** for 10 min at 4 °C, washed twice with sterile PBS and fixed with 6 ml of 70% (vol/vol) 2-propanol. Analysis of the polar lipids was then carried out by DSMZ Services, Leibniz-Institut DSMZ – Deutsche Sammlung von Mikroorganismen und Zellkulturen GmbH, Braunschweig, Germany. Both strains exhibited a polar lipid profile that was consistent with the *in silico* predictions and typical for other *Rothia* species, with the exception of increased DPG and aminoglycolipid (AGL) and decreased PG relative to other reported *Rothia* species (Fig. 4) [7,10,54, 56].

In conclusion, strains RSM42^T^, RSM292, RSM386 and RSM407 are members of a novel species of the genus *Rothia*, for which we propose the name *Rothia similimucilaginosa*. RSM42^T^ is the type strain. Based on phylogenetic analysis, phylogenomic analysis and the tests included in Tables1, 2  4, the novel species can be distinguished from its near relatives of the genus *Rothia*.

## PROTOLOGUE

### Description of *Rothia similimucilaginosa* sp. nov.

*Rothia similimucilaginosa* [si.mi.li.mu.ci.la.gi.no’sa. L. masc. adj. *similis*, similar; N.L. fem. adj. *mucilaginosa*, forming slimy colonies, and the specific epithet of a *Rothia* species; N.L. fem. adj. *similimucilaginosa*, similar to (*Rothia*) *mucilaginosa*].

Cells are Gram-stain-positive, coccoid-shaped and may produce extracellular polymeric substances. On TSA, cream-coloured, mucoid, non-translucent colonies are produced, with a diameter of ~1 mm. Colonies adhere to the agar surface, especially after 48 h of growth. Facultative anaerobic, with good growth aerobically, but poor growth anaerobically. Catalase-positive. Good growth occurs on nutrient-rich media, including BHI and TSA. On TSA, growth is observed at temperatures between 30 and 37 °C; weak growth is observed at 25, 40 and 45 °C; and growth is not observed at 20 °C or below. In addition, the strain grows well in a pH range of 6–8 but weakly at pH 9 and 10. When using the API Coryne test system, positive reactions are observed for: nitrate reduction, pyrrolidonyl arylamidase, α-glucosidase and fermentation of d-glucose, d-maltose and sucrose. Negative reactions are observed for: pyrazinamidase, alkaline phosphatase, β-glucuronidase, β-galactosidase, *N*-acetyl-β-glucosaminidase, aesculin hydrolysis, urease, gelatin hydrolysis and fermentation of d-ribose, d-xylose, d-mannitol, d-lactose and glycogen. When using the API ZYM test system, positive reactions are observed for: leucine arylamidase, valine arylamidase, naphthol-AS-BI-phosphatase and α-glucosidase. Negative reactions are observed for: alkaline phosphatase, C4 esterase, C8 esterase, C14 lipase, cystine arylamidase, trypsin, α-chymotrypsin, acid phosphatase, α-galactosidase, β-galactosidase, β-glucuronidase, β-glucosidase, *N*-acetyl-β-glucosaminidase, α-mannosidase and α-fucosidase. With Biolog GP2, positive reactions are observed for: acetoacetic acid, dextrin, d-fructose, d-fructose-6-phosphate, d-galacturonic acid, α-d-glucose, glycerol, glycyl-l-proline, α-keto-butyric acid, l-lactic acid, d-lactic acid methyl ester, d-maltose, d-mannose, pectin, d-serine, l-serine, sucrose, d-trehalose and d-turanose. Negative reactions are observed for: acetic acid, d-cellobiose, citric acid, d-fucose, l-fucose, l-galactonic acid lactone, d-galactose, gelatin, gentibiose, d-glucose-6-phosphate, d-glucuronic acid, l-glutamic acid, d-melibiose, 3-methyl glucose, β-methyl-d-glucoside, propionic acid, l-rhamnose and d-salicin. The major fatty acids (>10%) are anteiso-C_15:0_ and iso-C_16:0_. The moderate fatty acids are iso-C_14:0_, iso-C_15:0_, C_16:0_ and anteiso-C_17 : 0_. The major polar lipids are DPG and AGL.

The type strain of the novel species, *R. similimucilaginosa*, is strain RSM42^T^, isolated from the nasal cavity of an 8-year-old male from Madison, Wisconsin, in Spring 2008. RSM42^T^ has been deposited in the American Type Culture Collection (ATCC TSD-447^T^) and the Leibniz Institute DSMZ (DSM 118581^T^). The 16S rRNA gene sequence and the draft genome sequence of RSM42^T^ have been deposited in GenBank under accession numbers PQ549674 and GCA_029850985.1, respectively. The full genome sequence has been submitted to GenBank, and the accession is currently pending. The genome is 2.09 Mb with a DNA G+C content of 58.4 mol%, and it encodes 1,739 predicted genes.

## Supplementary material

10.1099/ijsem.0.007024 Uncited Supplementary Material 1.

## Abbreviations

AAI: average amino acid identity
AGL: aminoglycolipid
ANI: average nucleotide identity
ANIb: BLAST+ average nucleotide identity
ANIm: MUMmer3 average nucleotide identity
ATCC: American Type Culture Collection
BGC: biosynthetic gene cluster
BHI: brain heart infusion
CARD rgi: Comprehensive Antibiotic Resistance Database Resistance Gene Identifier
CLSI: Clinical and Laboratory Standards Institute
dDDH: digital DNA-DNA hybridization
DPG: diphosphatidylglycerol
DSM: DSMZ-German Collection of Microorganisms and Cell Culture
KEGG: Kyoto Encyclopaedia of Gene and Genomes
ML: maximum-likelihood
MP: maximum parsimony
NaCl: Sodium Chloride
NJ: neighbour-joining
PG: phosphatidylglycerol
TSA: Tryptone Soy Agar
TSB: Tryptone Soy Broth
