# Supplementary material for: Rothia similimucilaginosa sp. nov., isolated from the human nasal cavity
Source: Int J Syst Evol Microbiol. 2026 Jan 16;76(1):007024. doi: 10.1099/ijsem.0.007024 (PMC12811043; doi:10.1099/ijsem.0.007024)
Supplement: Uncited Supplementary Material 1. [file ijsem-76-07024-s001.pdf]

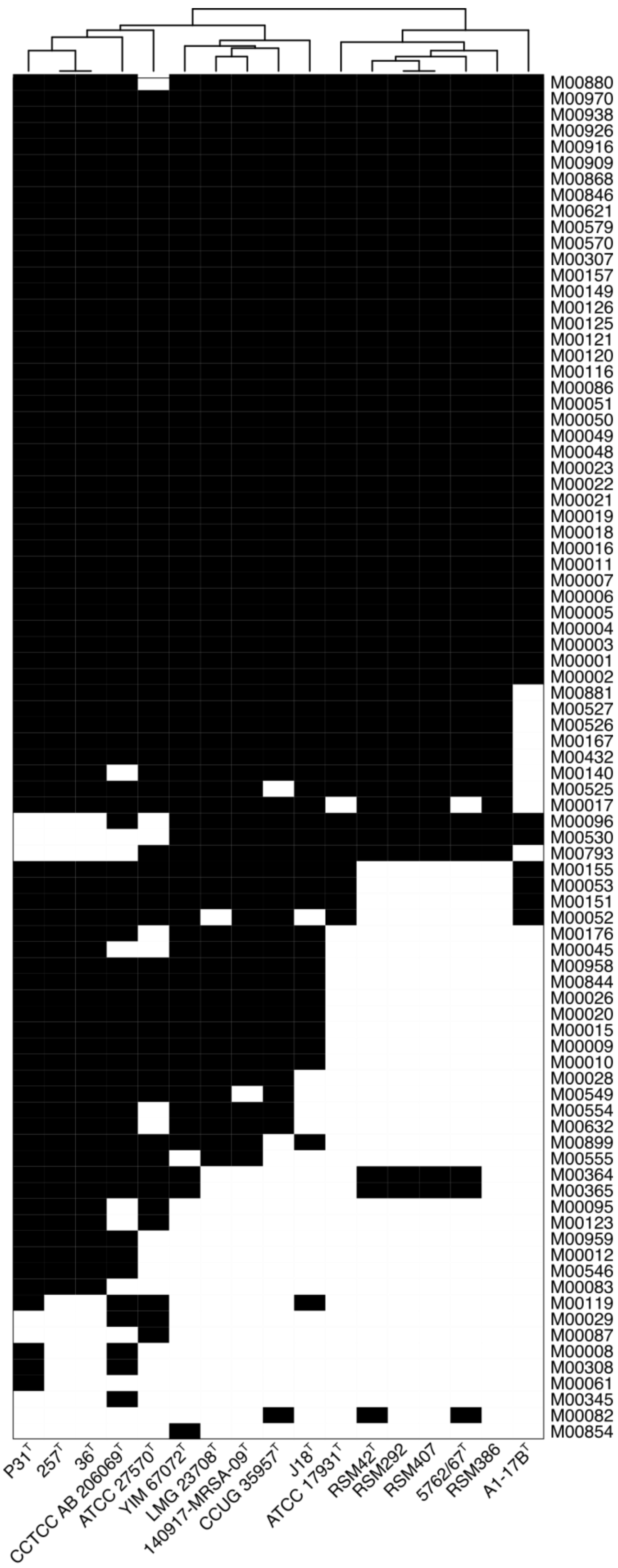

Figure S1. KEGG modules in *Rothia*. Heat map of KEGG modules (rows) predicted in *Rothia* genomes (columns). The presence or absence of a KEGG module in a given genome is indicated by black or white, respectively.

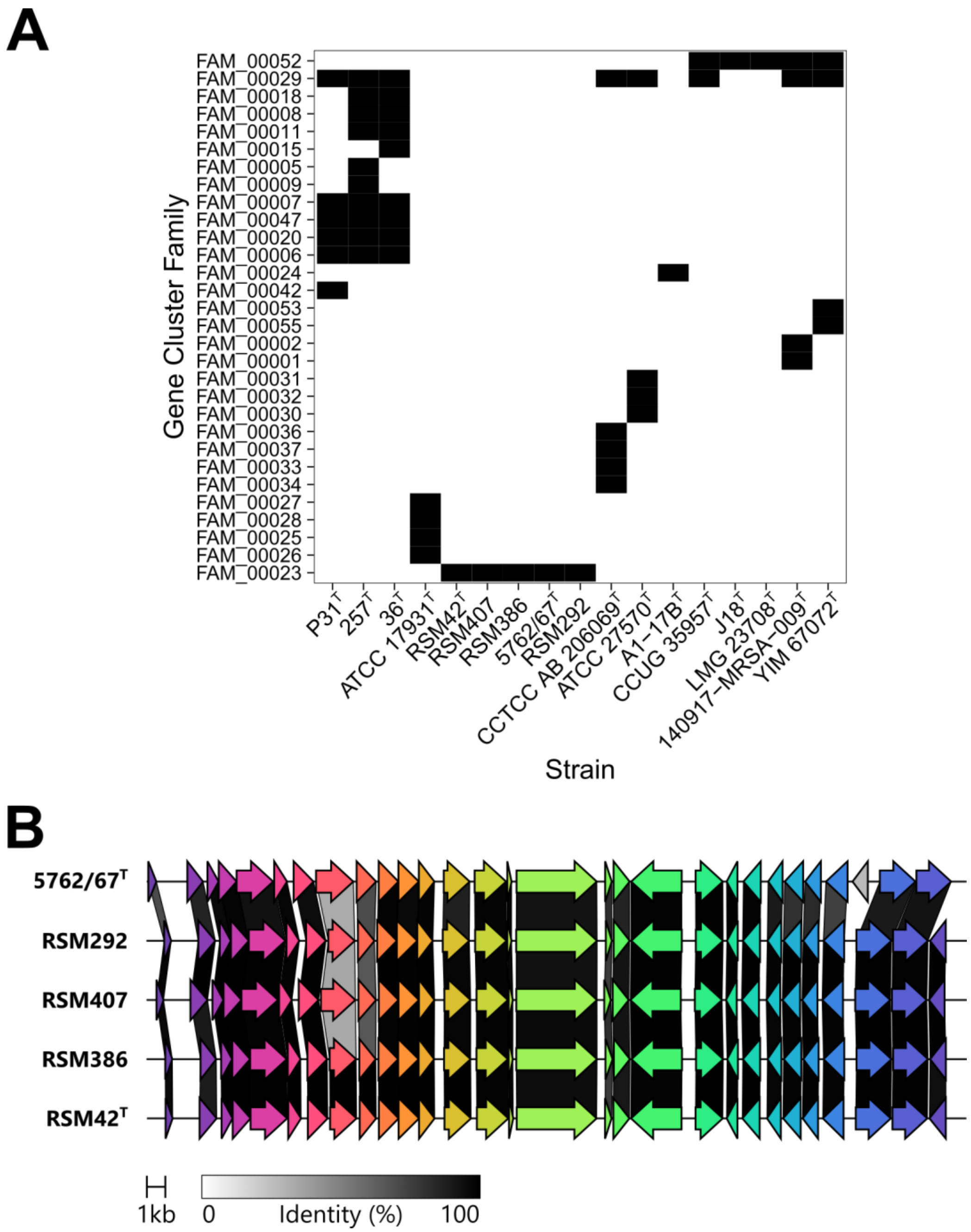

Figure S2. Secondary metabolite biosynthetic gene clusters in *Rothia*. (**A**) Heat map of secondary metabolite BGC families (rows) predicted in *Rothia* genomes (columns). The presence or absence of a BGCs in a given genome is indicated by black or white, respectively. (**B**) Synteny of the enterobactin BGC (FAM_00023) in *R. mucilaginosa* 5762/67^{T} and RSM42^{T}, RSM292, RSM387, and RSM407. The scale bar indicates the size of the genomic regions. Shared colors indicate homologous genes. Connections between genes represent sequence identity, as indicated by the scale bar at the bottom.

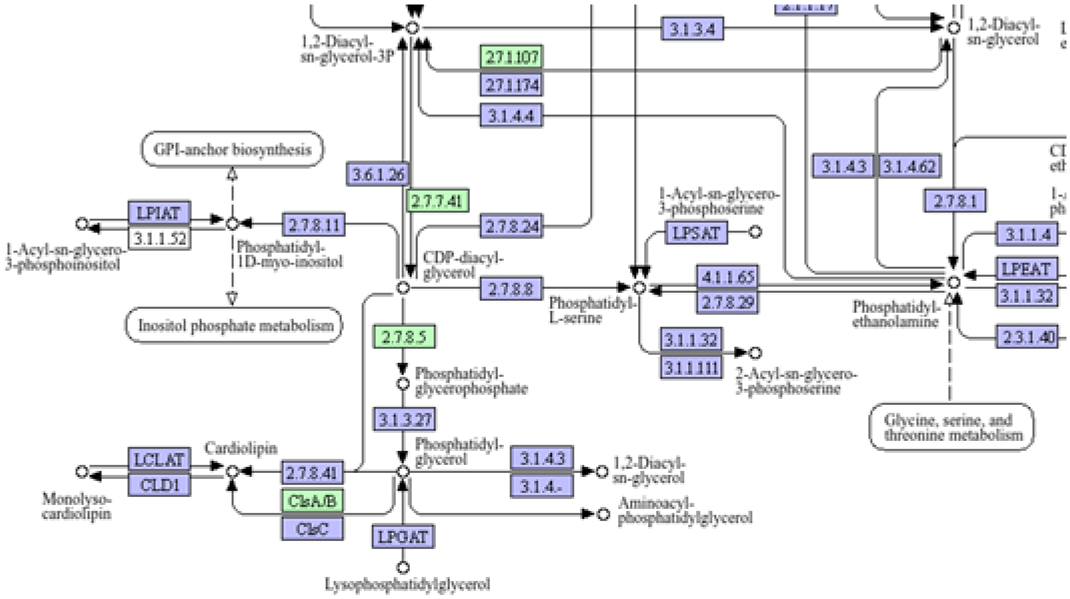

Figure S3. Glycerophospholipid metabolism of strain RSM42^{T} as determined from KEGG. Predicted products are phosphatidylglycerol and diphosphatidyglycerol.

Table S1. Genome assembly statistics for *Rothia* species used in this study. Strains: 1, RSM42^T; 2, RSM292; 3, RSM386; 4, RSM407; 5, *R. mucilaginosa* 5762/67^T; 6, *R. dentocariosa* ATCC 17931^T; 7, *R. aeria* A1-17B^T; 8, *R. amarae* J18^T; 9, *R. aerolata* 140917-MRSA-09^T; 10, *R. terrae* LMG 23708^T; 11, *R. endophytica* YIM 67072^T; 12, *R. nasimurium* CCUG3 5957^T; 13, *R. halotolerans* CCTCC AB 206069^T; 14, *R. kristinae* ATCC 27570^T; 15, *R. uropygialis* 36^T; 16, *R. uropygioeca* 257^T; 17, *R. koreensis* P31^T.

	**1**	**2**	**3**	**4**	**5**	**6**	**7**	**8**	**9**
Size (bp)	2,081,824	2,054,257	2,046,814	2049,163	2,272,572	2,506,025	2,588,680	2,340,577	2,462,803
Contigs	32	25	20	23	2	1	1	21	7
N50	214,071	173,089	212,831	174,143	2,165,174	2,506,025	2,588,680	228,729	1,379,322
L50	4	4	4	4	1	1	1	4	1
GC (%)	58.4	58.5	58.5	58.5	59.5	53.7	56.8	52.2	57.9
Accession	GCA_0298 50985.1	GCA_0298 50915.1	GCA_0298 50875.1	GCA_0298 50835.1	6d106fed5 6314878[a]	GCA_0001 64695.2	GCA_0023 55935.1	GCA_0395 25765.1	GCA_0146 35585.1

	**10**	**11**	**12**	**13**	**14**	**15**	**16**	**17**
Size (bp)	2,482,397	2,502,701	2,489,170	3,037,640	2,362,630	2,963,259	2,889,347	2,911,929
Contigs	41	30	35	10	14	10	6	44
N50	122,067	1,461,627	134,887	1,054,027	419,088	735,507	868,323	118,248
L50	7	1	5	2	2	2	2	6
GC (%)	53.4	56.3	59.9	71.5	71.9	61.1	60.8	64.5
Accession	GCA_0123 96615.1	GCA_0395 43885.1	GCA_0426 60305.1	GCA_0041 36635.1	GCA_0041 36565.1	GCA_0041 37765.1	GCA_0041 36585.1	GCA_0041 36575.1

[a]Available from the ATCC Website: https://genomes.atcc.org/genomes/6d106fed56314878

Table S2. ANIb comparisons for RSM42^T, RSM292, RSM386, RSM407, and all other validly published *Rothia* spp.

	**P31^T**	**257^T**	**36^T**	**ATCC 27570^T**	**CCTCC AB 206069^T**	**CCUG 35957^T**	**YIM 67072^T**	**LMG 23708^T**	**140917-MRSA-09^T**	**J18^T**	**A1-17B^T**	**ATCC 17931^T**	**5762/67^T**	**RSM386**	**RSM292**	**RSM407**	**RSM42^T**
**P31^T**	100	78.1	78.0	75.2	75.9	73.1	72.3	72.2	72.9	71.6	72.5	72.1	73.5	73.1	73.2	73.1	73.1
**257^T**		100	93.5	73.9	74.7	72.5	71.7	72.0	72.2	71.4	72.1	71.9	72.8	72.8	72.7	72.8	72.8
**36^T**			100	74.2	74.7	72.7	72.0	72.3	72.6	71.7	72.2	71.9	73.0	72.7	72.7	72.6	72.7
**ATCC 27570^T**				100	78.3	74.3	73.1	72.5	74.0	71.8	73.1	72.3	74.3	74.0	73.9	74.0	73.9
**CCTCC AB 206069^T**					100	74.3	73.0	72.5	73.8	71.9	73.0	72.6	74.7	74.2	74.3	74.3	74.3
**CCUG 35957^T**						100	76.9	74.7	76.0	73.6	74.3	73.7	75.9	75.6	75.6	75.6	75.6
**YIM 67072^T**							100	74.8	74.7	73.7	73.2	73.3	74.7	74.6	74.5	74.6	74.6
**LMG 23708^T**								100	74.8	74.3	73.3	73.5	74.6	74.6	74.6	74.6	74.6
**140917-MRSA-09^T**									100	74.4	73.9	73.9	75.5	75.2	75.2	75.2	75.2
**J18^T**										100	72.8	72.8	73.8	73.7	73.6	73.7	73.7
**A1-17B^T**											100	81.5	76.8	76.5	76.5	76.6	76.5
**ATCC 17931^T**												100	76.2	76.0	76.0	75.9	76.0
**5762/67^T**													100	91.4	91.3	91.4	91.4
**RSM386**														100	98.4	98.5	98.5
**RSM292**															100	98.6	98.5
**RSM407**																100	98.5
**RSM42^T**																	100

Table S3. ANIm comparisons for RSM42^T, RSM292, RSM386, RSM407, and all other validly published *Rothia* spp.

	**P31^T**	**257^T**	**36^T**	**ATCC 27570^T**	**CCTCC AB 206069^T**	**CCUG 35957^T**	**YIM 67072^T**	**LMG 23708^T**	**140917-MRSA-09^T**	**J18^T**	**A1-17B^T**	**ATCC 17931^T**	**5762/67^T**	**RSM386**	**RSM292**	**RSM407**	**RSM42^T**
**P31^T**	100	84.3	84.1	84.5	84.6	84.2	84.5	85.3	84.4	86.5	86.1	83.2	83.8	85.8	85.6	85.5	85.6
**257^T**		100	93.7	84.5	84.7	84.8	85.9	86.4	84.8	88.6	86.2	86.8	84.1	86.6	86.5	86.6	86.4
**36^T**			100	84.7	84.6	84.4	85.3	86.6	84.5	87.9	85.5	85.0	83.6	86.8	86.0	86.8	86.5
**ATCC 27570^T**				100	85.1	83.5	84.4	86.1	83.2	89.1	85.6	82.7	83.3	84.4	84.5	84.5	84.4
**CCTCC AB 206069^T**					100	83.4	85.0	85.6	83.3	88.6	84.6	83.7	83.1	84.3	84.6	84.4	84.4
**CCUG 35957^T**						100	85.4	84.4	84.1	84.6	84.3	84.1	83.9	84.4	84.4	84.3	84.5
**YIM 67072^T**							100	86.3	84.9	85.7	85.0	85.7	84.4	85.2	84.9	85.0	85.3
**LMG 23708^T**								100	85.5	86.6	85.1	86.3	84.4	85.1	85.2	85.1	85.1
**140917-MRSA-09^T**									100	85.6	84.6	85.2	84.0	84.9	84.9	84.9	85.0
**J18^T**										100	84.6	85.5	85.2	85.5	85.9	85.8	85.6
**A1-17B^T**											100	90.8	84.8	85.1	85.0	85.1	85.2
**ATCC 17931^T**												100	85.1	85.2	85.4	85.4	85.3
**5762/67^T**													100	92.0	91.9	92.0	92.0
**RSM386**														100	98.5	98.5	98.6
**RSM292**															100	98.6	98.5
**RSM407**																100	98.6
**RSM42^T**																	100

Table S4. dDDH comparisons for RSM42^T, RSM292, RSM386, RSM407, and all other validly published *Rothia* spp.

	**P31^T**	**257^T**	**36^T**	**ATCC 27570^T**	**CCTCC AB 206069^T**	**CCUG 35957^T**	**YIM 67072^T**	**LMG 23708^T**	**140917-MRSA-09^T**	**J18^T**	**A1-17B^T**	**ATCC 17931^T**	**5762/67^T**	**RSM386**	**RSM292**	**RSM407**	**RSM42^T**
**P31^T**	100	20.6	20.3	20.2	19.9	21.3	21.0	21.7	20.1	20.2	23.2	23.7	22.1	21.8	21.8	21.7	21.9
**257^T**		100	52.9	20.1	20.2	21.7	20.6	21.8	22.4	22.4	22.9	23.1	24.0	22.7	22.4	22.7	23.3
**36^T**			100	20.1	19.7	21.1	19.9	20.9	22.1	22.5	23.8	23.4	23.6	23.8	23.8	23.7	24.1
**ATCC 27570^T**				100	21.4	20.6	21.4	22.2	20.6	22.7	24.2	24.0	22.1	21.7	22.1	22.0	22.2
**CCTCC AB 206069^T**					100	20.1	20.6	21.0	20.6	20.9	22.0	22.4	21.3	20.7	20.5	20.5	21.3
**CCUG 35957^T**						100	22.0	22.6	21.5	22.5	21.8	21.6	23.0	22.5	22.5	22.6	22.7
**YIM 67072^T**							100	24.1	21.6	22.0	21.3	24.6	23.2	22.8	23.1	22.7	22.9
**LMG 23708^T**								100	21.7	23.2	23.6	24.4	23.1	22.9	22.7	22.9	23.0
**140917-MRSA-09^T**									100	21.5	21.7	24.2	22.8	23.0	23.0	23.0	22.9
**J18^T**										100	22.7	23.6	23.4	24.0	25.4	25.3	25.3
**A1-17B^T**											100	32.1	23.8	23.6	23.4	23.4	23.5
**ATCC 17931^T**												100	24.1	23.9	23.8	23.7	23.7
**5762/67^T**													100	43.5	43.1	43.3	43.3
**RSM386**														100	85.9	86.4	86.8
**RSM292**															100	87.5	86.3
**RSM407**																100	87.0
**RSM42^T**																	100

Table S5. Phenotypic characteristics of RSM292, RSM386, and RSM407. Strains: 1, RSM292; 2, RSM386; 3, RSM407. All data were obtained under identical conditions. +, Positive, −, Negative, (+), Weak positive reaction; ND, no data.

**Characteristic**	**1**	**2**	**3**
Colony morphology	cream, mucoid	cream, mucoid	cream, mucoid
Cell morphology	Gram-stain-positive, coccoid, width: 1.3 µm	Gram-stain-positive, coccoid, width: 1.4 µm	Gram-stain-positive, coccoid, width: 1.5 µm
Optimal Growth temperature (range)	25 – 37°C (weak at 20°C or 40°C)	25 – 37°C (weak at 20°C or 40°C)	25 – 37°C (weak at 20°C or 40°C)
Optimal Growth pH (range)	6 – 8 (weak at 9 – 10)	6 – 8 (weak at 9 – 10)	6 – 8 (weak at 9 – 10)
NaCl tolerance (range, %)	0.5 – 4.5 (weak at 5.5%)	0.5 – 4.5 (weak at 5.5%)	0.5 – 4.5 (weak at 5.5%)
DNA G+C Content (%)	58.4	58.5	58.4
Enzyme activities:			
Catalase	+	+	+
C4 Esterase	−	−	−
β-Glucosidase	−	−	−
Pyrazinamidase	−	−	−
Trypsin	−	−	−
Valine Arylamidase	+	+	+
Reactions:			
Gelatin hydrolysis	−	+	+
Nitrate reduction	+	+	+

Table S6. Carbon source utilization by RSM292, RSM386, and RSM407. Strains: 1, RSM292; 2, RSM386; 3, RSM407. +, Positive, −, Negative, (+), Weak positive reaction.

**Carbon source**	**1**	**2**	**3**
Dextrin	+	+	+
D-maltose	+	+	+
D-trehalose	+	+	+
D-cellobiose	−	−	−
Gentibiose	(+)	−	−
Sucrose	+	+	+
D-turanose	+	+	+
D-melibiose	(+)	(+)	−
β-methyl-D-glucoside	+	(+)	+
D-salicin	+	(+)	+
α-D-glucose	+	+	+
D-mannose	+	+	+
D-fructose	(+)	+	+
D-galactose	+	+	(+)
3-methyl glucose	(+)	+	(+)
D-fucose	+	+	(+)
L-fucose	+	+	(+)
L-rhamnose	+	+	−
D-serine	−	−	(+)
Glycerol	+	+	+
D-glucose-6-phosphate	(+)	(+)	−
D-fructose-6-phosphate	+	+	+
Gelatin	+	−	−
Glycyl-L-proline	+	+	+
L-glutamic acid	−	−	−
L-serine	+	(+)	−
Pectin	+	+	+
D-galacturonic acid	+	+	−
L-galactonic acid lactone	+	+	(+)
D-glucuronic acid	+	+	−
D-lactic acid methyl ester	(+)	(+)	−
L-lactic acid	+	+	+
Citric acid	−	−	−
α-keto-butyric acid	+	+	(+)
Acetoacetic acid	+	+	+
Propionic acid	−	−	−
Acetic acid	−	−	−

Table S7. Strict antimicrobial resistance gene (AMRs) detected by the Comprehensive Antibiotics Resistance Database Resistance Gene Identifier (CARD rgi) in RSM42^T and other *Rothia* species. Strains: 1, RSM42^T; 2, *R. mucilaginosa* 5762/67^T; 3, *R. dentocariosa* ATCC 17931^T; 4, *R. aeria* A1-17B^T; 5, *R. amarae* J18^T; 6, *R. aerolata* 140917-MRSA-09^T; 7, *R. terrae* LMG 23708^T; 8, *R. endophytica* YIM 67072^T; 9, *R. nasimurium* CCUG3 5957^T; 10, *R. halotolerans* CCTCC AB 206069^T; 11, *R. kristinae* ATCC 27570^T; 12, *R. uropygialis* 36^T; 13, *R. uropygioeca* 257^T; 14, *R. koreensis* P31^T. +, Positive, −, Negative, (+).

**Gene**	**AMR Gene Family**	**1**	**2**	**3**	**4**	**5**	**6**	**7**	**8**	**9**	**10**	**11**	**12**	**13**	**14**
AAC(6')-Ie-APH(2'')-Ia	aminoglycoside bifunctional resistance protein	−	−	−	−	−	−	+	−	−	−	−	−	−	−
ermX	Erm 23S ribosomal RNA methyltransferase	−	−	−	−	−	−	−	+	−	−	−	−	−	−
qacG	small multidrug resistance (SMR) antibiotic efflux pump	−	−	−	−	+	+	−	+	−	−	−	−	−	−
qacJ	small multidrug resistance (SMR) antibiotic efflux pump	−	−	−	−	−	−	+	−	+	−	−	−	−	−
rpsL	antibiotic-resistant *rpsL*	−	−	−	−	−	+	−	−	−	−	−	−	−	−
vanW	*vanW*, glycopeptide resistance gene cluster	−	−	−	−	−	−	+	−	−	−	+	−	−	−
vanY	*vanY,* glycopeptide resistance gene cluster	+	+	+	+	−	+	−	+	+	+	+	−	−	−

Table S8. Cellular fatty acid profiles of RSM292, RSM386, and RSM407. Strains: 1, RSM292; 2, RSM386; 3, RSM407. Fatty acids are listed in order of increasing retention time. All data were obtained under identical conditions. Fatty acids with ≥10% relative abundance are shown in bold.

**Fatty acid**	**1**	**2**	**3**
$C_{12:0}$	0.5	0.5	0.5
anteiso-$C_{13:0}$	4.8	3.0	1.6
iso-$C_{14:0}$	6.4	5.7	3.8
$C_{14:0}$	2.2	2.1	2.2
iso-$C_{15:0}$	5.9	5.5	5.6
anteiso-$C_{15:0}$	**52.4**	**52.7**	**46.1**
$C_{15:0}$	0.8	1.2	1.1
iso-$C_{16:0}$	**10.6**	**12.1**	**13.5**
$C_{16:0}$	7.6	6.9	9.9
iso-$C_{17:0}$	0.5	0.5	0.9
anteiso-$C_{17:0}$	7.1	8.4	**12.9**
